# Morphological and phylogenetic evidence reveal three new *Pseudohydnum* (*Auriculariales*, Basidiomycota) species from North China

**DOI:** 10.3389/fcimb.2023.1139449

**Published:** 2023-02-15

**Authors:** Hong-Min Zhou, Tolgor Bau, Jing Si

**Affiliations:** ^1^ Institute of Microbiology, School of Ecology and Nature Conservation, Beijing Forestry University, Beijing, China; ^2^ Engineering Research Center of Chinese Ministry of Education for Edible and Medicinal Fungi, Jilin Agricultural University, Changchun, China

**Keywords:** taxonomy, phylogeny, hydnoid fungi, gelatinous fungi, temperate forests, species diversity

## Abstract

*Pseudohydnum* is characterized by gelatinous basidiomata with hydnoid hymenophores and longitudinally septate basidia. In this study, samples of the genus from North China were examined morphologically and phylogenetically using a dataset of the internal transcribed spacer of the ribosomal RNA gene and the nuclear large subunit rDNA. This study describes three new species, namely *Pseudohydnum abietinum*, *Pseudohydnum candidissimum*, and *Pseudohydnum sinobisporum*. *Pseudohydnum abietinum* is characterized by pileate and pale clay pink basidiomata when fresh, with a rudimentary stipe base, four-celled basidia, and broadly ellipsoid to ovoid or subglobose basidiospores (6−7.5 × 5−6.3 μm). *P. candidissimum* is characterized by very white basidiomata when fresh, frequently four-celled basidia, and broadly ellipsoid to subglobose basidiospores (7.2−8.5 × 6−7 μm). *P. sinobisporum* is characterized by ivory basidiomata when fresh, two-celled basidia, ovoid to broadly ellipsoid, or subglobose basidiospores (7.5−9.5 × 5.8−7.2 μm). The main characteristics, type localities, and hosts of *Pseudohydnum* species are listed.

## 1 Introduction

*Pseudohydnum* P. Karst., typified by *P. gelatinosum* (Scop.) P. Karst. (Karsten, 1868), has high nutritional and medicinal values (Wang, 2012; Wu et al., 2019). The genus belongs to *Auriculariales* and is characterized by gelatinous basidiomata with conical spines, a monomitic hyphal system with clamp connections on generative hyphae, longitudinally septate basidia, and ovoid to ellipsoid or globose basidiospores (Niveiro and Popoff, 2011; Chen et al., 2020). Unlike the transversely septate (auricularioid) basidia, the genus has longitudinally cruciate-septate (tremellioid) basidia and thus was treated in *Tremellales* (Karsten, 1868; Breitenbach and Kränzlin, 1986; Courtecuisse and Lowy, 1990; Niveiro and Popoff, 2011). However, Ingold (1982; 1985) noted that *Pseudohydnum* and *Exidia* have a relatively close relationship based on spore germination. Morphologically, Bandoni (1984) redefined the concept of *Auriculariales*, and the family *Aporpiaceae* was used to accommodate taxa with myxarioid basidia, including *Pseudohydnum*. Weiss and Oberwinkler (2001) verified that *Pseudohydnum* has a close relationship with *Auriculariales* based on phylogenetic analyses; however, the position of *Pseudohydnum* in *Auriculariales* was ambiguous.

Eight species have been recognized in *Pseudohydnum*. The type species *P. gelatinosum* was found in Europe (Scopoli, 1772), and two varieties, *P. gelatinosum* var. *bisporum* Lowy & Courtec. and *P. gelatinosum* var. *paucidentatum* Lowy, were discovered in North America (Lowy, 1959, 1971; Courtecuisse and Lowy, 1990). Three species were described from Oceania: *P. orbiculare* J.A. Cooper, *P. tasmanicum* Y.C. Dai & G.M. Gates, and *P. totarae* (Lloyd) J.A. Cooper (Zhou et al., 2022). Four species were described from Asia: *P. translucens* Lloyd, *P. brunneiceps* Y.L. Chen et al., *P. himalayanum* Y.C. Dai et al., and *P. sinogelatinosum* Y.C. Dai et al., of which the last three species were described from China (Chen et al., 2020; Zhou et al., 2022). In addition, two forms, *P. gelatinosum* f. *album* (Bres.) Kobayasi and *P*. *gelatinosum* f. *fuscum* (Bres.) Kobayasi, have been described from Japan (Lloyd, 1925; Kobayasi, 1954).

During an investigation of jelly fungi in North China, several samples belonging to *Pseudohydnum* were collected, and three unknown species were found. To confirm the affinity of the taxa, phylogenetic analysis was performed based on the internal transcribed spacer (ITS) and large subunit nuclear ribosomal RNA gene (LSU) sequences.

## 2 Materials and methods

### 2.1 Morphological studies

The specimens were collected from the provinces of Jinlin, Heilongjiang, and Gansu in North China. They were deposited in the herbaria of Beijing Forestry University (BJFC) and the Mycology Department of Jinlin Agriculture University (HMJAU). Samples were photographed when fresh in the field, and their habitats were recorded. Microscopic structures were discussed by Chen et al. (2020), Fan et al. (2021), and Zhou et al. (2022). Special color terms were set by Anonymous (1969) and Petersen (1996). A Nikon Digital Sight DS-L3 or Leica ICC50 HD camera (magnification ×1,000) was used to examine hand-cut sections of basidiomata, which were first treated with 5% KOH for a few minutes and then with 1% phloxine B (C_20_H_4_Br_4_Cl_2_K_2_O_5_). At least 30 basidiospores of each species were examined. The values were expressed as a mean with 5% of the measurements excluded from each end of the range, given in parentheses. Stalks were excluded for basidia measurement, and the hilar appendages were excluded for basidiospore measurement.

The following abbreviations are used in the descriptions: IKI, Melzer’s reagent; IKI−, neither amyloid nor dextrinoid; CB, cotton blue; CB−, acyanophilous in cotton blue; L, the arithmetic average of spore lengths; W, the arithmetic average of spore widths; Q, L/W ratio; and n (a/b), number of spores (a) measured from a given number (b) of specimens.

### 2.2 DNA extraction, amplification, and sequencing

The CTAB rapid plant genome extraction kit-DN14 (Aidlab Biotechnologies Co., Ltd., Beijing) was used to obtain DNA from dried specimens and PCR was performed according to the manufacturer’s instructions with some modifications (Chen and Dai, 2021). Two DNA gene fragments, ITS and LSU, were amplified using the primer pairs ITS5/ITS4 (White et al., 1990) and LR0R/LR7, respectively (Hopple and Vilgalys, 1994).

The PCR procedure for ITS was as follows: initial denaturation at 95 °C for 3 min, followed by 35 cycles at 94 °C for 40 s, 54 °C for 45 s, and 72 °C for 1 min; and a final extension at 72 °C for 10 min. The PCR procedure for LSU was as follows: initial denaturation at 94 °C for 1 min, followed by 35 cycles at 94 °C for 30 s, 50 °C for 1 min, and 72 °C for 1.5 min; and a final extension at 72 °C for 10 min. DNA sequencing was performed at the Beijing Genomics Institute. All newly generated sequences were submitted to GenBank and are listed in **Table 1**.

**Table 1:** Taxa information and GenBank accession numbers of the sequences used in this study.

Species	Locality	Voucher	ITS	LSU
***Pseudohydnum abietinum***	**China**	**Dai 24185**	**OP965350**	**OP965370**
***P. abietinum***	**China**	**Dai 24194**	**OP965351**	**OP965371**
*Pseudohydnum brunneiceps*	China	JXSB 0967	MN497254	MN497259
*P. brunneiceps*	China	JXSB 1063	MN497257	MN497258
***Pseudohydnum candidissmum***	**China**	**Dai 23740**	**OP965345**	**OP965365**
***P. candidissmum***	**China**	**HMJAU 5312**	**OP965346**	**OP965366**
***P. candidissmum***	**China**	**HMJAU 23836**	**OP965347**	**OP965367**
*Pseudohydnum gelatinosum*	China	Dai 21665	ON243826	ON243924
*P. gelatinosum*	Denmark	DMS-9327933	MT644890	MT644890
*P. gelatinosum*	Germany	MW 298	DQ520094	DQ520094
*P. gelatinosum*	UK	K(M): 250843	MZ159722	–
“*P. gelatinosum*-1”	Canada	ANT 187-QFB 28623	MN992495	–
“*P. gelatinosum*-1”	Canada	ANT 017-QFB 28581	MN992496	–
“*P. gelatinosum*-2”	Canada	UBC: F19746	HQ604801	HQ604801
“*P. gelatinosum*-2”	USA	S.D. Russell MycoMap # 1379	MK575262	–
*Pseudohydnum himalayanum*	China	Cui 17045	ON243829	ON243927
*P. himalayanum*	China	Cui 17065	ON243830	ON243928
*Pseudohydnum orbiculare*	New Zealand	PDD 112653	ON243832	–
*P. orbiculare*	New Zealand	PDD 112654	ON24383	ON243929
***Pseudohydnum sinobisporum***	**China**	**HMJAU 33728**	**OP965349**	**OP965369**
***P. sinobisporum***	**China**	**SYL 2307**	**OP965348**	**OP965368**
*Pseudohydnum sinogelatinosum*	China	Cui 17064	ON243833	–
*P. sinogelatinosum*	China	Cui 17074	ON243834	ON243930
*Pseudohydnum tasmanicum*	Australia	Cui 16721	ON243838	ON243934
*P. tasmanicum*	Australia	Dai 18724	ON243839	ON243935
*Pseudohydnum totarae*	New Zealand	PDD 96246	ON243840	–
*P. totarae*	New Zealand	PDD 112652	ON243841	–
*P. totarae*	New Zealand	PDD 112655	ON243842	ON243936
*Protomerulius subreflexus*	Indonesia	OM 14402.1	MG757508	MG757508
*Protomerulius substuppeus*	Costa Rica	O 19171	JX134482	JQ764649

New sequences are in bold. The symbol "-" represents that there is no sequence.

Sequences generated for this study were aligned, with additional sequences downloaded from GenBank. Both ITS and LSU sequences were aligned using MAFFT v.7 (https://mafft.cbrc.jp/alignment/server/), adjusting the direction of nucleotide sequences according to the first sequence (accurate enough for most cases), and selecting the G-INS-i iterative refinement method (Katoh et al., 2019). Alignments were manually adjusted to maximize alignment and minimize gaps with BioEdit v.7.0.9 (Hall, 1999). A dataset composed of concatenated ITS + LSU sequences was used to determine the phylogenetic position of new species. The aligned sequences were deposited in TreeBase (https://www.treebase.org/treebase-web/home.html; submission ID 29962). *Protomerulius subreflexus* (Lloyd) O. Miettinen & Ryvarden and *P*. *substuppeus* (Berk. & Cooke) Ryvarden were selected as outgroups following Chen et al. (2020).

Maximum likelihood (ML) analysis was performed using the CIPRES Science Gateway (Miller et al., 2009) based on the dataset using the RA × ML-HPC BlackBox tool, with setting RA × ML halt bootstrapping automatically and 0.25 for maximum hours and obtaining the best tree using ML search. Other parameters in ML analysis used default settings, and statistical support values were obtained using nonparametric bootstrapping with 1,000 replicates.

Bayesian inference (BI) analysis based on the dataset was performed using MrBayes v.3.2.6 (Ronquist et al., 2012). The best substitution model for the dataset was selected by ModelFinder (Kalyaanamoorthy et al., 2017) using a Bayesian information criterion, and the model was used for Bayesian analysis. Four Markov chains were run from random starting trees for 0.8 million generations. Trees were sampled every 1,000th generation. The first 25% of sampled trees were discarded as burn-in, whereas other trees were used to construct a 50% majority consensus tree and for calculating Bayesian posterior probabilities (BPPs).

## 3 Results

### 3.1 Phylogeny

**Figure 1:**
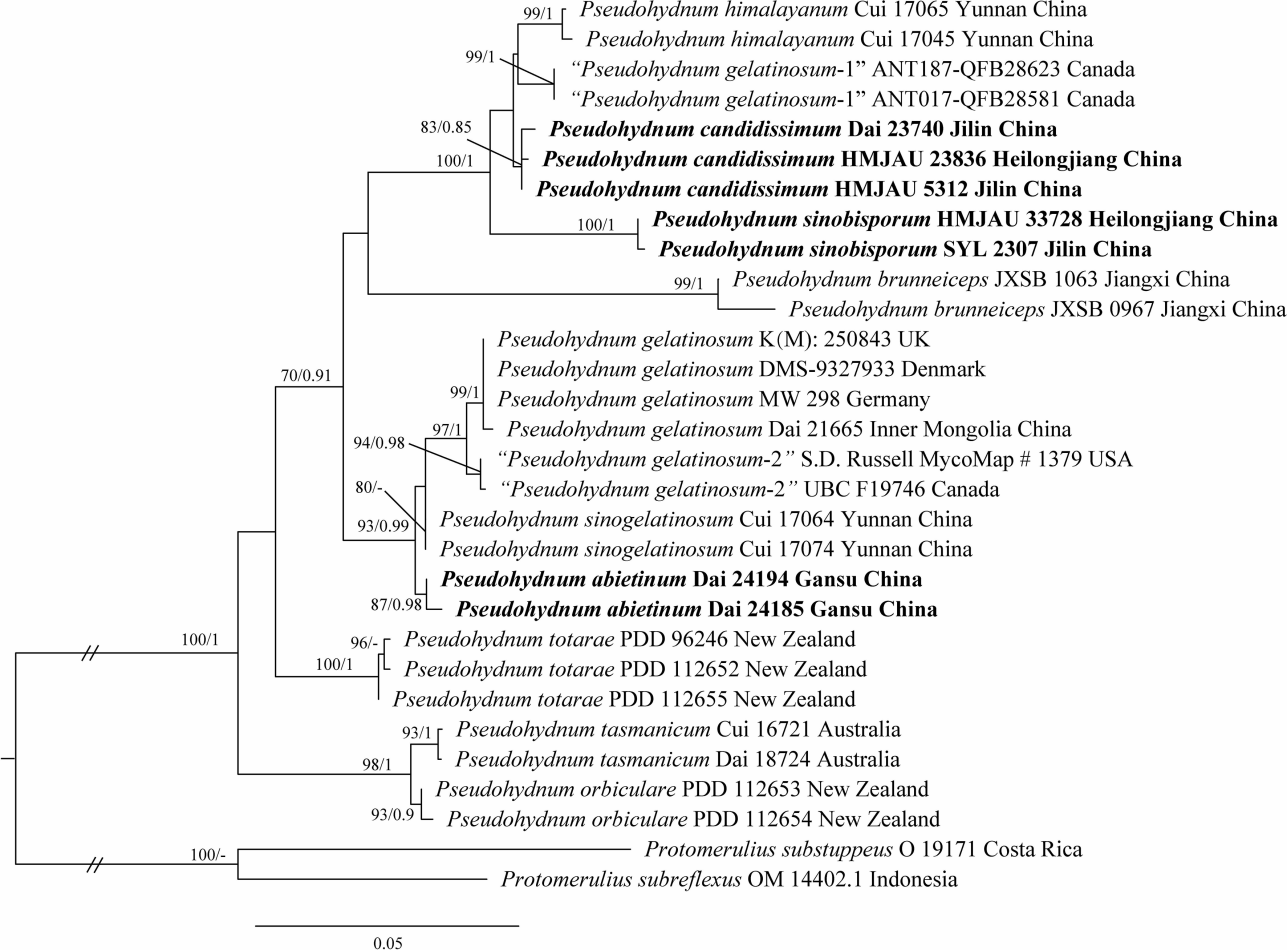
Phylogeny of *Pseudohydnum* species generated by maximum likelihood (ML) based on ITS + LSU sequences. Branches are labeled with ML bootstrap ≥70% and Bayesian posterior probabilities ≥0.85.

The concatenated ITS + LSU dataset included 30 ITS and 22 LSU sequences from 30 samples representing 14 taxa. The best model for the concatenated ITS + LSU dataset estimated and applied for BI analysis was “SYM + I + G”, datatype = DNA, nucmodel = 4by4, lset nst = 6, rates = invgamma; state frequencies had a Dirichlet prior (1,1,1,1), and the distribution was approximated using four categories. BI analysis yielded a similar topology to ML analysis, with an average standard deviation of split frequencies of 0.007485. The ML tree was provided (**Figure 1**). Branches that received bootstrap support for ML (ML-BS) and BI (BPP) ≥70% (ML-BS), and 0.85 (BPP) were considered significantly supported, respectively.

Phylogenetic analysis placed all *Pseudohydnum* samples in a fully supported clade (100/1, **Figure 1**). Five specimens from Northeast China formed two lineages, namely *P. candidissmum* and *P. sinobisporum*, clustered with *P. himalayanum* as strong support (100/1, **Figure 1**). The two specimens from Northwest China were named *P. abietinum*, sister to *P. sinogelatinosum* and *P. gelatinosum*. The samples from North America were treated as “*P. gelatinosum*-1” and “*P. gelatinosum*-2.”

### 3.2 Taxonomy

**Figure 2:**
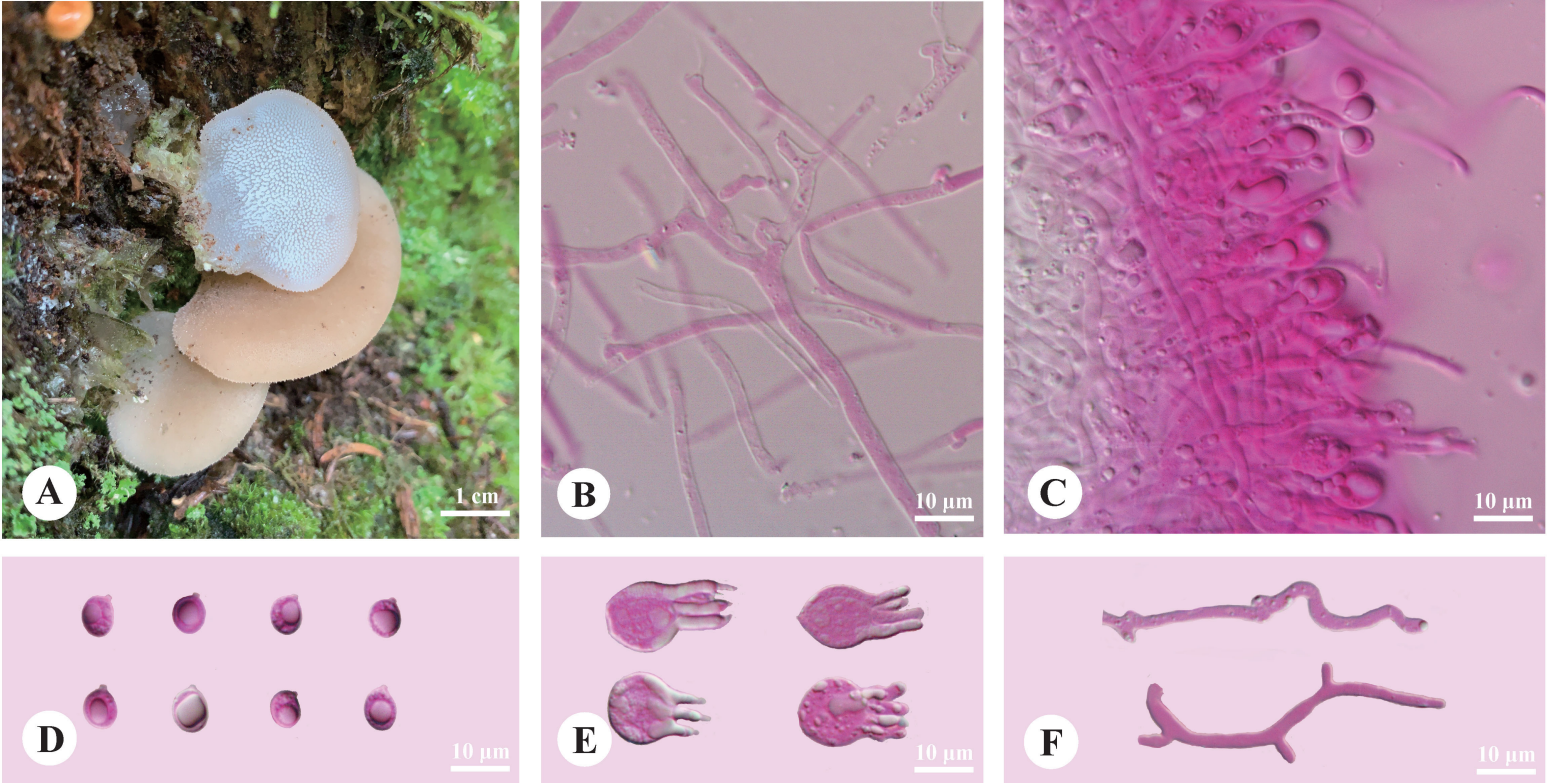
Basidiomata and microscopic structures of *Pseudohydnum abietinum* (holotype, Dai 24185). **(A)** Basidiomata; **(B)** Tramal hyphae; **(C)** A section of hymenium; **(D)** Basidiospores; **(E)** Basidia; **(F)** Hyphidia.

*Pseudohydnum abietinum* H.M. Zhou & Jing Si, sp. nov. **Figure 2**

MycoBank: 847486

Type—China. Gansu Province, Gannan, Zhuoni County, Taohe National Nature Reserve, Boyu Valley, on a stump of *Abies*, elev. 2,900 m, August 19, 2022, Dai 24185 (holotype, BJFC).

Etymology—*Abietinum* (Lat.): referring to the species growing on *Abies*.

Diagnosis—Differed from other *Pseudohydnum* species in having pileate basidiomata, with a rudimentary stipe base, pale clay pink pileal surface when fresh, hymenophore with spines 2−3 per mm at the base, broadly ellipsoid to ovoid or subglobose basidiospores measuring 6−7.5 × 5−6.3 μm, and occurring in Gansu Province, Northwest China.

Basidiomata—Annual, pileate with a rudimentary stipe base, gelatinous when fresh, brittle when dry, usually solitary. Pilei were dimidiated to flabelliform, projecting up to 1.5 cm, 1.4 cm wide, and 1.9 mm thick when dry. Pileal surfaces were pale clay pink when fresh, and hazel when dry. Spines were white and conical when fresh, cream when dry, 2−3 per mm at the base, and up to 1.5-mm long when dry. The context was translucent when fresh.

Hyphal structure—Monomitic; generative hyphae with clamp connections. Contextual hyphae were hyaline, thin- to slightly thick-walled, frequently branched, interwoven, and 2−6 μm in diam. Tramal hyphae were hyaline, thin-walled, frequently branched, interwoven, and 1.5−2 μm in diam. Hyphidia were occasionally branched. Basidia were four-celled, barrel-shaped, globose to subglobose, with guttules, and 9.5−12 × 7.5−12 μm; sterigmata were up to 12-μm long and 1.5−2 μm in diam. Probasidia were fusiform to lageniform and 10−13 × 8−10.5 μm. Basidiospores were broadly ellipsoid to ovoid or subglobose, hyaline, thin-walled, with a big guttule, IKI−, CB−, 6−7.5(−8) × 5−6.3(−6.8) μm, L = 6.84 μm, W = 5.59 μm, and Q = 1.20−1.24 (60/2).

Additional specimen examined (paratype)—China. Gansu Province, Gannan, Zhuoni County, Taohe National Nature Reserve, Boyu Valley, on rotten wood of *Abies*, elev. 2,900 m, August 19, 2022, Dai 24194 (BJFC).

**Figure 3:**
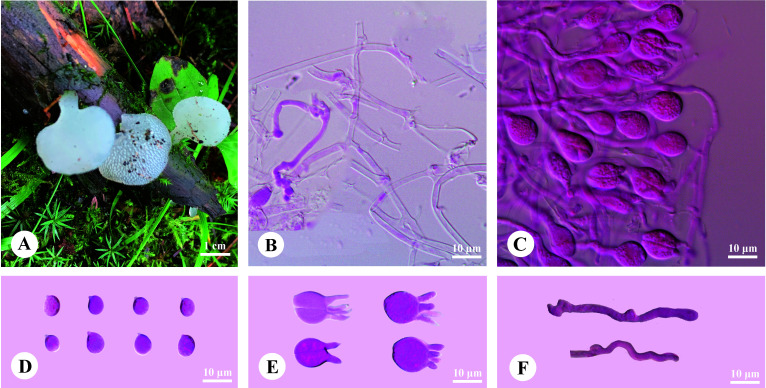
Basidiomata and microscopic structures of *Pseudohydnum candidissimum* (holotype, Dai 23740). **(A)** Basidiomata; **(B)** Tramal hyphae; **(C)** A section of hymenium; **(D)** Basidiospores; **(E)** Basidia; **(F)** Hyphidia.

*Pseudohydnum candidissimum* H.M. Zhou, T. Bau & Jing Si, sp. nov. **Figure 3**

Mycobank: 847487

Type—China. Jilin Province, Yanbian, Antu County, Changbaishan Nature Reserve, on a fallen trunk of *Larix*, July 24, 2022, Dai 23740 (holotype, BJFC).

Etymology—*Candidissimum* (Lat.): referring to the species having very white basidiomata when fresh.

Diagnosis—Differed from other *Pseudohydnum* species in having very white basidiomata when fresh, simple hyphidia, broadly ellipsoid to subglobose, measuring 7.2−8.5 × 6−7 μm, and occurring in Northeast China.

Basidiomata—Annual, gelatinous when fresh, brittle when dry, usually solitary, with a lateral stipe. Pilei flabelliform to dimidiate, projecting up to 1.5 cm, 1.2 cm wide, and 0.6-mm thick when dry. The pileal surface was white when fresh and pale mouse-gray when dry. Spines were white and conical when fresh, buff-yellow when dry, 2−3 per mm at the base, and up to 0.5-mm long. The context was translucent when fresh. Stipe concolorous with pileal surface, translucent when fresh, up to 5-mm long and 3 mm in diam. when dry.

Hyphal structure—Monomitic; generative hyphae with clamp connections. Contextual hyphae were hyaline, thin- to slightly thick-walled, frequently branched, interwoven, 1.5−3 μm in diam. Tramal hyphae were hyaline, thin-walled, frequently branched, interwoven, 1.5−2 μm in diam. Hyphidia simple. Basidia were frequently four-celled, occasionally two-celled, barrel-shaped, ellipsoid to subglobose, 11−14 × 10.5−13 μm; sterigmata up to 10-μm long and 2−3.5 μm in diam. Probasidia were fusiform to lageniform, 11−14 × 6.5−10 μm. Basidiospores were broadly ellipsoid to subglobose, hyaline, thin-walled, IKI−, CB−, (7−)7.2−8.5(−9.2) × 6−7(−7.5) μm, L = 7.97 μm, W = 6.56 μm, and Q = 1.19−1.24 (90/3).

Additional specimens examined (paratypes)—China. Heilongjiang Province, Yichun, Fenglin National Nature Reserve, July 25, 2010, HMJAU 23836; Jilin Province, Yanbian, Antu County, Erdaobai River, on rotten wood, September 13, 2007, HMJAU 5312.

**Figure 4:**
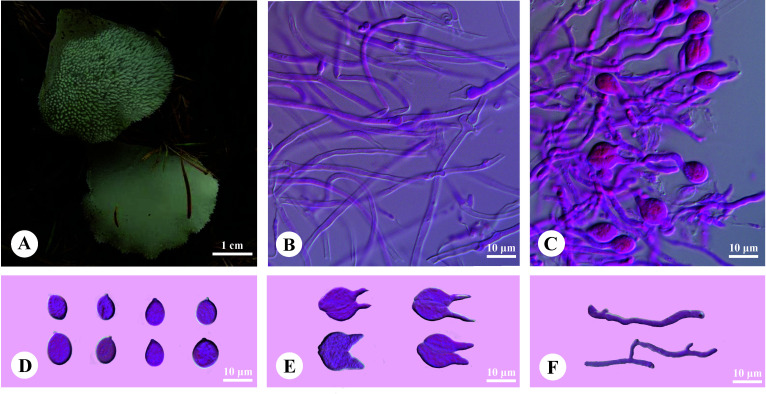
Basidiomata and microscopic structures of *Pseudohydnum sinobisporum* (holotype, SYL 2307). **(A)** Basidiomata; **(B)** Tramal hyphae; **(C)** A section of hymenium; **(D)** Basidiospores; **(E)** Basidia; **(F)** Hyphidia.

*Pseudohydnum sinobisporum* T. Bau, H.M. Zhou & Jing Si, sp. nov. **Figure 4**

MycoBank: 847488

Type—China. Jilin Province, Yanbian, Tianfozhishan National Nature Reserve, on a stump of *Quercus mongolica*, August 23, 2020, SYL 2307 (holotype, HMJAU).

Etymology—*Sinobisporum* (Lat.): referring to the species having two spores on each basidium and being found in China.

Diagnosis—Differed from other *Pseudohydnum* species in having ivory basidiomata, two spores on each basidium, branched hyphidia, ovoid to broadly ellipsoid or subglobose, measuring 7.5−9.5 × 5.8−7.2 μm, and occurring in Northeast China.

Basidiomata—Annual, gelatinous when fresh, brittle when dry, solitary, with a lateral stipe. Pilei was shell-shaped, projecting up to 1.2 cm, 1 cm wide, and 1.2 mm thick when dry. Pileal surfaces were ivory when fresh and hazel when dry. Spines were white and conical when fresh, cream when dry, 2−3 per mm at the base, and up to 1 mm long when dry. The context was translucent when fresh. Stipe concolorous with pileal surface, shrinking to the base, translucent when fresh, up to 5.5-mm long and 5 mm in diam. when dry.

Hyphal structure—Monomitic; generative hyphae with clamp connections. Contextual hyphae were hyaline, thin- to slightly thick-walled, frequently branched, interwoven, and 1.5−3 μm in diam. Tramal hyphae were hyaline, thin-walled, occasionally branched, interwoven, and 1−2 μm in diam. Hyphidia were occasionally branched. Basidia were two-celled, barrel-shaped, ellipsoid to subglobose, 11−11.5 × 9−12 μm; sterigmata were up to 11-μm long and 2−3 μm in diam. Probasidia were fusiform to lageniform, 11−15 × 8−11.5 μm. Basidiospores were ovoid to broadly ellipsoid or subglobose, hyaline, thin-walled, IKI−, CB−, (7.2−)7.5−9.5(−10) × (5.5−)5.8−7.2(−7.5) μm, L = 8.29 μm, W = 6.36 μm, Q = 1.30−1.31 (60/2).

Additional specimen examined (paratype)—China. Heilongjiang Province, Tahe County, on the ground in the forest of *Larix*, August 19, 2015, HMJAU 33728.

## 4 Discussion

Morphological examination and phylogenetic analysis identified eight species of *Pseudohydnum* (Chen et al., 2020; Zhou et al., 2022). In this study, three new species of *Pseudohydnum* were identified in North China: *P. abietinum*, *P. candidissimum*, and *P. sinobisporum*.

Phylogenetically, *P. abietinum* formed a sister group with *P. gelatinosum*, *P. sinogelatinosum*, and “*P. gelatinosum*-2” (**Figure 1**). However, *P. gelatinosum* had smaller basidiospores than *P. abietinum* (5−6 × 4.5−5.5 μm vs. 6−7.5 × 5−6.3 μm, Breitenbach and Kränzlin, 1986), and *P. sinogelatinosum* had wider basidiospores than *P. abietinum* (6−7.2 μm vs. 5−6.3 μm, Zhou et al., 2022) (**Table 2**). Samples of “*P*. *gelatinosum*-2” were not evaluated in this study.

**Table 2:** A comparison of the morphologies, type localities, and hosts of *Pseudohydnum* species.

Taxa	Type locality	Pileal surface when fresh	Spines size at the base (per mm)	Stipe	Basidiospores (μm)	Basidia (μm)	Hosts	References
***Pseudohydnum abietinum***	**China**	**Pale clay pink**	**2−3**	**Absent**	**6−7.5 × 5−6.3**	**9.5−12 × 7.5−12, 4**-**celled**	***Abies***	**This study**
*Pseudohydnum brunneiceps*	China	Pale yellowish brown, dark reddish brown to blackish	−	Present	6−8 × 5.5−7	9−13 × 6−10, 2−4-celled	*Cryptomeria*	Chen et al. (2020)
***Pseudohydnum candidissimum***	**China**	**White**	**2−3**	**Present**	**7.2−8.5 × 6−7**	**11−14 × 10.5−13, 2−4**-**celled**	***Larix***	**This study**
*Pseudohydnum gelatinosum*	Croatia	White	5−7	−	5−6 × 4.5−5.5	10−11.5 × 12−13, 4-celled	*Larix*	Breitenbach and Kränzlin (1986); this study
*Pseudohydnum gelatinosum* var. *bisporum*	French Guiana	Beige-grayish	−	−	5.5−8.5 × 5.5−7.5	10−12, 2-celled	Rotten wood	Courtecuisse and Lowy (1990)
*Pseudohydnum gelatinosum* var. *paucidentatum*	Bolivia	White	−	−	7−9 × 6−8.5	12.5−15 × 9−11, 2−4-celled	Dicot wood	Lowy (1959)
*Pseudohydnum himalayanum*	China	Clay-pink to cinnamon	5−6	Present	7−8.5 × 6−7.2	12−17.5 × 6−13.5, 4-celled	*Abies*	Zhou et al. (2022)
*Pseudohydnum orbiculare*	New Zealand	White to grayish brown to reddish brown	0.5−1	Absent	6.5−7.9 × 5.6−6.8	10−14 × 10, 4-celled	−	Zhou et al. (2022)
***Pseudohydnum sinobisporum***	**China**	**Ivory**	**2−3**	**Present**	**7.5−9.5 × 5.8−7.2**	**11−11.5 × 9−12, 2**-**celled**	***Quercus* and *Larix***	**This study**
*Pseudohydnum sinogelatinosum*	China	Pinkish buff to cinnamon-buff	3−4	Present	7−9 × 6−7.2	12−15 × 10−12, 4-celled	*Pinus*, *Abies*, and *Picea*	Zhou et al. (2022)
*Pseudohydnum tasmanicum*	Australia	Light vinaceous gray to smoke gray	2−3	Absent	7.2−9 × 6−7.2	12−15 × 10−11, 2−4-celled	*Eucalyptus* and *Nothofagus*	Zhou et al. (2022)
*Pseudohydnum totarae*	New Zealand	White to grayish brown to reddish brown	0.8−1.2	Present	5.5−6.5 × 4.8−5.7	9−13 × 8, 4-celled	*Podocarpus*, *Agathis*, and *Dacrydium*	Zhou et al. (2022)
*Pseudohydnum translucens*	Japan	Pure white	−	−	4−5 × 4−5	−	−	Lloyd (1925)

New species are in bold. The symbol "-" represents that there is no sequence.

*Pseudohydnum candidissimum* and *P. sinobisporum* were related to *P. himalayanum* and “*P. gelatinosum*-1” (**Figure 1**); however, *P. himalayanum* had denser spines at the base (5−6 per mm vs. 2−3 per mm, Zhou et al., 2022) and was clay-pink to cinnamon basidiomata when fresh (**Table 2**). Samples of “*P*. *gelatinosum*-1” were not evaluated in this study.

Morphologically, *P. himalayanum* and *P. abietinum* had similar basidiomata and were easily confused; however, *P. himalayanum* had wider basidiospores than *P. abietinum* (6−7.2 µm vs. 5−6.3 µm, Zhou et al., 2022). *Pseudohydnum tasmanicum* and *P. abietinum* shared a rudimentary stipe; however, *P. tasmanicum* had wider basidiospores than *P. abietinum* (6−7.2 µm vs. 5−6.3 µm, Zhou et al., 2022).

Similar to *P. candidissimum*, *P. gelatinosum* and *P. gelatinosum* var. *paucidentatum* had white basidiomata (**Figure 3**; **Table 2**); however, *P. gelatinosum* had smaller basidiospores than *P. candidissimum* (5−6 × 4.5−5.5 μm vs. 7.2−8.5 × 6−7 μm, Breitenbach and Kränzlin, 1986). Compared to *P. candidissimum*, *P. gelatinosum* var. *paucidentatum* had widely scattered spines, the color of its basidiomata remained unchanged upon drying (Lowy, 1959, 1971), and it is distributed in tropical America.

*Pseudohydnum brunneiceps*, *P. gelatinosum* var. *bisporum*, and *P. sinobisporum* had two-celled basidia (**Figure 4**; **Table 2**). However, *P. brunneiceps* had brownish basidiomata and occurs in subtropical China; *P. gelatinosum* var. *bisporum* had short elliptical to subglobose basidiospores and is distributed in French Guiana and South America (Courtecuisse and Lowy, 1990), whereas the newly discovered *P. sinobisporum* had ivory basidiomata, which were ovoid to broadly ellipsoid or subglobose, and is distributed in boreal to temperate China.

*Pseudohydnum candidissimum* and *P. sinobisporum* had overlapping distributions in Northeast China; however, *P*. *candidissimum* had very white basidiomata and mostly four-celled basidia (**Figure 3**; **Table 2**), and *P*. *sinobisporum* had ivory basidiomata and two-celled basidia (**Figure 4**; **Table 2**).

Jelly fungi are a special group of wood-inhabiting basidiomycetes and most species belong to the taxa form phragmobasidia (Wells, 1994). Most belong to *Auriculariales* and *Tremellales*, and some are edible mushrooms (Dai et al., 2010; Luo et al., 2022; Yao et al., 2022; Zhang et al., 2022). However, the diversity of the Chinese jelly fungi is not well-known, and recently, new species were described from China based on both morphology and phylogeny (Wu et al., 2020, 2021; Fan et al., 2021; Zhou et al., 2022). Advanced techniques, including molecular phylogeny and omics, will aid in discovering new species of jelly fungi in the future.

## Data availability statement

The datasets presented in this study can be found in online repositories. The names of the repository/repositories and accession number(s) can be found below: https://www.ncbi.nlm.nih.gov/genbank/, OP965345-OP965351 and https://www.ncbi.nlm.nih.gov/genbank/, OP965365-OP965371.

## Author contributions

Design of the research: H-MZ, TB, and JS. Performance of the research: H-MZ and JS. Data analysis and interpretation: H-MZ and JS. Collection of materials: H-MZ and TB. Writing and revising the manuscript: H-MZ, JS, and TB. All authors listed have made a substantial, direct, and intellectual contribution to the work and approved it for publication.
